# Perinatal depression as a risk-factor for infant sleep disturbances: Subjective data from a case-control study

**DOI:** 10.1192/j.eurpsy.2021.1477

**Published:** 2021-08-13

**Authors:** M. Caccialupi O. P., C. Pucci, M. Colaiori, N. Giacchetti, F. Aceti, C. Sogos

**Affiliations:** Human Neuroscience, University of Rome - La Sapienza, Rome, Italy

**Keywords:** Perinatal depression, sleep disorders, Mother-child interaction, Relational changes

## Abstract

**Introduction:**

Perinatal period is characterized by a broad range of physical, psychological and relational changes. Maternal perinatal depression (PD) is defined as an episode of major depression with the onset from pregnancy to the first year after delivery. Depressive symptoms influence the earlier mother-child interaction and impact on child cognitive, affective and behavioral development.

**Objectives:**

Purpose of our study was to evaluate the consequences of PD on sleep-wake patterns in the early stages of infant development. We aim to investigate the presence of poor sleep in infants/ toddlers and also to identify differences in sleep ecology variables.

**Methods:**

We enrolled, from December 2019 to September 2020, a clinical sample of children born from women with PD (N=19, m.a.=13,7, SD= 7,6) and a healthy control group (N=21, m.a.=15,5, SD=5,43). Infant sleep data were obtained from the Brief Infant Sleep Questionnaire (BISQ). Poor sleepers were defined by the following criteria: >3 night wakings, nocturnal wakefulness >1 hr or total sleep duration <9 hr. Maternal depression was assessed with clinical and psychometric evaluation. T-test was used for comparison between the two samples.

**Results:**

Statistical analysis indicates that there were not significant differences between the two groups concerning night wakings (p= .678), nocturnal wakefulness (p= .815), total sleep duration (p= .209) and nocturnal sleep onset time (p= .475).

**Conclusions:**

Our findings suggest that PD is not a risk-factor in the onset of infant sleep problems. Probably negative parentig, affective disengagement, delegation in maternal care and sedative effects of pharmacotherapy may affect mother’s perception of her infant’s sleep.

